# Case Report and Literature Review: Behçet’s Disease With a Novel TFPI Gene Mutation

**DOI:** 10.3389/fmed.2022.873600

**Published:** 2022-04-19

**Authors:** Jiewen Ma, Wengang Sun, Liang Tang, Di Yang

**Affiliations:** Institute of Hematology, Union Hospital, Tongji Medical College, Huazhong University of Science and Technology, Wuhan, China

**Keywords:** thrombosis, Behçet’s disease, TFPI, mutation, case report

## Abstract

We report a case of Behçet’s disease (BD) with a newly identified tissue factor pathway inhibitor (TFPI) gene mutation. The patient suffered from recurrent deep vein thrombosis and dural sinus thrombosis which could not be relieved by constant anticoagulation therapy. Slight relapsing oral lesion was the initial manifestation of BD but was neglected. Genital ulcers and ocular symptoms were manifest 8-month later than vascular involvement. The patient was diagnosed with BD at last and a novel mutation in TFPI was identified simultaneously. After administration with azathioprine and dexamethasone, the clinical symptoms were quickly gone and no relapse was found during 7-month follow-up.

## Introduction

Behçet’s disease (BD) is a chronic multisystemic vasculitis which affects both arteries and veins with non-specific inflammation. The clinical symptoms of BD are protean and commonly characterized with oral aphthae, genital ulcerations and ocular involvement. Though the etiology of BD remains uncertain, many factors have been thought contributory, including geographic region, genetic variants, viruses’ infection and so on (1, 2). Its prevalence and clinical manifestations also show regional differences (3). BD is most frequent from the Mediterranean region to the Far East, and gastrointestinal involvement is more commonly described in these areas (4–6). The onset age of BD is usually in the third decade, with significant morbidity and mortality reported to be associated with precocious onset, particularly in male patients.

Vascular involvement affects approximately 15 – 40% BD patients, and 27.5% of these patients may exhibit vascular lesion as their initial manifestation (7). Vascular complications before classic symptoms of BD or meeting the International Criteria for Behçet’s disease (ICBD) greatly increase the difficulty in diagnosis. Deep vein thrombosis (DVT) is most common and mainly found in lower extremities. Venous thrombosis in other sites includes vena cava and dural sinus thrombosis. It was also reported that dural sinus thrombosis was significantly associated with systemic major vessel disease (8).

Tissue factor pathway inhibitor (TFPI) is a vital modulator in coagulation cascade. On one hand, it forms a quaternary complex with tissue factor, activated factor VII and X. On the other hand, it binds with activated factor V, inhibiting the conversion from prothrombin to thrombin (9). The majority of TAPI is bound to vessel endothelial cells, and only 20 – 50% circulates in blood (10). Low levels of plasma TFPI are reported to increase the risk of thrombosis in both veins and arteries (11). Genetic variants may contribute to the heritable variation of TAFI level in plasma or affects its interaction with other cofactors (12–14).

Here we report a case in which a young man suffered from recurrent thrombosis and eventually identified with BD and a novel mutation in TFPI. The double hit of widespread vasculitis and disturbed modulating of coagulation may explain the refractory thrombophilia. To our knowledge, the mutation carried by this patient is newly identified and this is the first reported case presenting recurrent thrombosis caused by BD with thrombophilic genetic mutation. The patient and his family were fully informed and written consents were signed. This work is approved by the ethics committee of Union Hospital at Huazhong University of Science and Technology.

## Case Presentation

**FIGURE 1:**
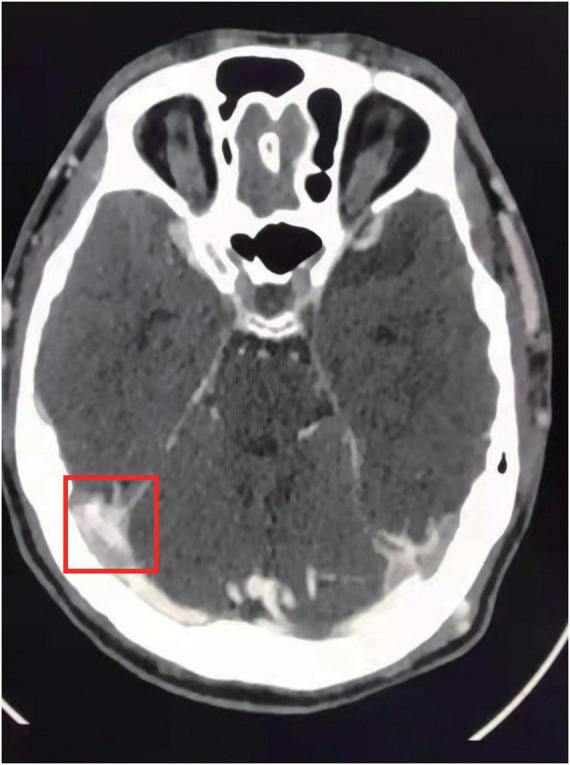
MRI scan indicated dural sinus thrombosis.

A 21-year-old Chinese male patient who had suffered from recurrent thromboembolism events for nearly 1 year was admitted to our hospital. With no history of major health issues or family history regarding similar conditions reported, the patient exhibited swelling and pain in lower left limb 1 year ago. A proximal leg vein ultrasound scan indicated thrombosis and anticoagulation treatment was carried out with 2-week low molecular weight heparin (LMWH) and 8-month rivaroxaban. About 1 month after finished anticoagulation, he caught a cold and exhibited headache, vomiting and double vision in the left eye afterward. Cerebral angiography in local hospital indicated dural sinus thrombosis (Figure 1) and the patient was given anticoagulation with LMWH, dehydration therapy, neurotrophic treatment and so on. In the meantime, coagulation tests showed basically normal results. Nevertheless, after administration with LMWH for 50 days, the patient was diagnosed with DVT in lower right extremity again. Then the patient was transferred to our department.

**FIGURE 2:**
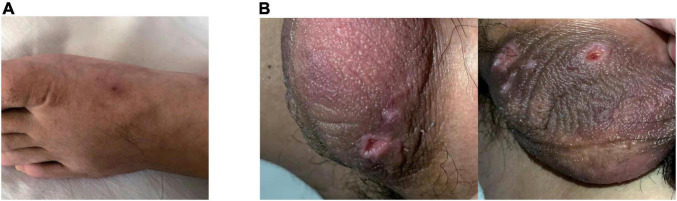
Erythema at injection sites when the patient was admitted **(A)**. Ulcerations on the scrotum were manifest 2-weeks later **(B)**.

In physical examination, limited abduction of his left eye, erythema at injection sites (Figure 2A) and raised temperature of skin on the right inner thigh were manifest, together with swelling of bilateral lower limbs, particularly in the right. Negative results were reported in investigations including extractable nuclear antigen, lupus anticoagulant and anti-cardiolipin antibodies. C-reactive protein (CRP) was 55 mg/L (normal: < 5 mg/L). Considering the refractory thrombi in deep veins of lower limbs and dural sinus, genetic screening for thrombophilia which used panel-based next generation sequencing (NGS) targeting genes involved in hemostasis, anticoagulation and fibrinolysis system was also applied. While waiting for the results, treatment with argatroban, dehydration, anti-infection and neurotrophic therapy were used. Two days after transferred to our department, laboratory data involved with coagulation testing, activities of coagulation factors showed prolonged APTT (64.5 s, normal: 20 – 47.1 s), TT (43.4 s, normal: 12.9 – 22.9 s) and reduction in activities of coagulation factors VIII (43%, normal: 70 – 150%), IX (51%, normal: 60 – 150%), XI (46%, normal: 70 – 120%) were observed. And treatment as described above for 2 weeks did not see release in right-leg swelling and the regional pain was even more severe. CRP consistently fluctuated between 50 and 60 mg/L.

**FIGURE 3:**
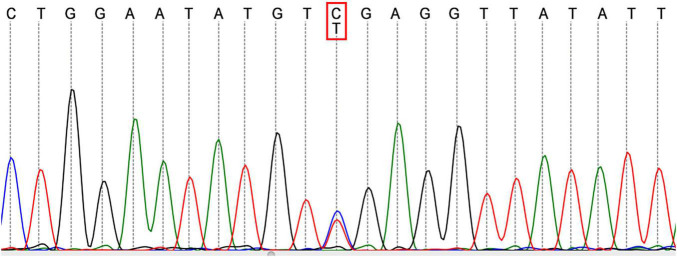
Sequencing analysis of the patient revealed a heterozygous mutation in TFPI.

Then the result of genetic screening came out, reporting a heterozygous mutation of TFPI (NM_006287; c.C403T: p.R135X; Figure 3). The variant was discovered by NGS and confirmed by Sanger sequencing. The variant of TFPI was also identified in his father but not his mother through further work-up. Meanwhile, the patient began to exhibit two ulcers on the scrotum (Figure 2B). But no oral aphthae were found with the patient. After repeated questioning, the patient reported recurrent genital ulcerations for half a year, and resolution always occurred spontaneously. He had gone to dermatologists and rheumatologists many times but no diagnosis had been made. There were also oral ulcerations three to four times a year for almost 2 years, along with occasional bloodshot eyes. Because of the mildness of these manifestations, he failed to put these signs together and did not mention them when admitted. According to the ICBD, the diagnosis of BD was made based on the oral and genital lesions, ocular involvement and vascular symptoms. The coexisting mutation in TFPI may also contribute to his refractory thrombophilia and resistance to anticoagulants.

## Treatment

After diagnosis of BD, azathioprine (100 mg qd), prednisone (4 mg qd) and continuous anticoagulation was administered to the patient.

## Outcome and Follow-Up

The patient promptly recovered and the swelling and localized pain in his right leg disappeared quickly. So did the ulcerations in scrotum. Reexamination showed a decrease to normal range in CRP. During our 7-month follow-up, the patient exhibited no relapse in thrombosis or ulcerations.

## Discussion

In this study, we reported a case characterized with recurrent thrombi in lower extremities and dural sinus. Vascular involvement was 8-month earlier than ocular symptoms and genital aphthae. Slight relapsing oral lesion was the initial manifestation. However, it failed to draw attention from the patient himself or the clinicians. Diverse symptom combinations and timeframe make the diagnosis of BD sometimes quite challenging. The patient was also identified with a novel mutation in TFPI, which may contribute to the refractory thrombophilia. The effect of continuous anticoagulation treatment was proved to be unsatisfactory in this case. For the most part, laboratory data of coagulation studies was basically normal, while the prolonged APTT, TT, and reduction in activities of coagulation factors VIII, IX, XI were probably caused by the administration of argatroban at that time. On the contrary, immunosuppressive agents and glucocorticoids were significantly effective. This case highlights the importance of a broad differential diagnosis when the patient suffers from recurrent multiple thrombi under unknown cause.

Behçet’s disease is classified as an inflammatory vascular disease affecting blood vessels of all sizes and kinds, which involves almost every organ and system. It is distributed globally, in spite of the regional differences of prevalence and manifestations. The most frequently described clinical symptom is mucocutaneous lesion. Oral aphthae is described in 98% of cases and genital aphthae in about 65% cases (15). While the spontaneously recovering ulcerations are quite negligible, diagnosis of BD is challenging -and relies on thorough medical history, which is indicated by our case.

The mechanism underlying vascular involvement in BD is not clear. Despite the fact that vascular syndrome is manifest in only approximately 15% patients, it contributes greatly to the morbidity and mortality of BD (16). Most BD patients with vascular involvement experience the first vascular event within 5 years since onset of the disease, which is earlier than fulfilling the diagnostic criteria in 10.8% of these patients (8). The primary objectives in treating BD patients with vascular involvement are suppressing vasculitis and preventing further complications (17). Standard guidelines regarding the management of thrombosis in BD patients are currently lacking. The first choice is immunosuppressive agents, while the usage of anticoagulants is controversial considering the risk of fatal bleeding when pulmonary arterial aneurysm is coexisting. A retrospective analysis of 807 BD patients showed 4-fold decreased thrombosis relapse by immunosuppressive agents alone (18). The relapse rates were similar when comparing patients using anticoagulants together with immunosuppressant or only immunosuppressant (19, 20). The usage of azathioprine and dexamethasone was effective in this case and follow-up of longer duration is needed to testify its effect on relapse. To reach reliable consensus and tailor for specific phenotypes of patients, more randomize controlled studies regarding management of vascular BD are needed.

Tissue factor pathway inhibitor is a vital anticoagulant which inhibits the earliest steps in extrinsic coagulation pathway. Threshold effect of low plasma TFPI levels on thrombotic risk has already been clarified. About two-fold increased risk of incident VTE and myocardial infraction was found in subjects with baseline TFPI levels in the lowest 5 – 10% of the distribution (21, 22). Dennis et al. (11) systematically summarized the genetic factors associated with levels of plasma TFPI. They found that the minor allele of rs5940 was related with decreased TFPI level, and mutations causing deficiencies in protein S, factor V, apolipoprotein E and galactosidase α also had influences on plasma TFPI level. Studies have reported controversial conclusions regarding the relation between some variants of TFPI and the caused risk for thrombosis (14, 23). The mutation C-403T identified in this patient is located in exons of TFPI, and we presume that this mutation has deleterious effects on anti-coagulation which needs further study to verify.

There are also obvious limitations in our study. How the TFPI variant affects the plasma level and modulatory function of TFPI is unclear. Further investigation is needed to clarify its importance. Follow-up study of the patient will help monitor recurrence or aggravation. This case elucidates the importance of early diagnosis and identifying the causes in choosing appropriate treatment. A comprehensive medical history and broad differential diagnosis are vital when the patient exhibits recurrent multiple thrombi under unknown cause.

## Learning Points

• The diagnosis of BD can be very challenging when vascular involvement is manifest earlier than the classical combinations of oral aphthae, genital ulcerations and ocular symptoms.
• A broad differential diagnosis and genetic screening for thrombophilia are vital when the patient exhibits recurrent multiple thrombi under unknown cause.
• A novel mutation in TFPI was identified in this case.

## Data Availability Statement

The original contributions presented in the study are included in the article/supplementary material, further inquiries can be directed to the corresponding author.

## Ethics Statement

Ethical Approval was provided by the Ethics Committee of Union Hospital at Huazhong University of Science and Technology. Written informed consent to participate was provided by the patient/next of kin. Written informed consent was obtained from the individual(s) for the publication of any potentially identifiable images or data included in this article.

## Author Contributions

LT and DY designed the study. DY and WS investigated and provided the clinical data. DY and JM wrote the manuscript. All authors contributed to the article and approved the submitted version.

## Conflict of Interest

The authors declare that the research was conducted in the absence of any commercial or financial relationships that could be construed as a potential conflict of interest.

## Publisher’s Note

All claims expressed in this article are solely those of the authors and do not necessarily represent those of their affiliated organizations, or those of the publisher, the editors and the reviewers. Any product that may be evaluated in this article, or claim that may be made by its manufacturer, is not guaranteed or endorsed by the publisher.
